# Case report of vasovagal syncope associated with single pulse transcranial magnetic stimulation in a healthy adult participant

**DOI:** 10.1186/s12883-015-0510-2

**Published:** 2015-12-01

**Authors:** Bernadette T. Gillick, Tonya Rich, Mo Chen, Gregg D. Meekins

**Affiliations:** University of Minnesota Medical School, Rehabilitation Science, 420 Delaware Street SE, MMC 388, Minneapolis, MN 55455 USA; Institute for Engineering in Medicine, University of Minnesota Non-Invasive Neuromodulation Laboratory, MnDRIVE Brain Conditions, 420 Delaware Street SE, MMC 388, Minneapolis, MN 55455 USA; University of Minnesota Medical School, Department of Neurology, Minneapolis, MN 55455 USA

## Abstract

**Background:**

Non-invasive brain stimulation-related seizures or syncopal events are rare. However, we report on a syncopal event in a healthy female during a transcranial magnetic stimulation single-pulse testing session.

**Case presentation:**

A 47-year-old healthy female presented for a transcranial magnetic stimulation session involving single-pulse assessment of cortical excitability. During the session, the participant appeared to have a brief event involving fainting and myoclonic jerks of the upper extremities. Orthostatic assessment was performed after the event and physician evaluation determined that this was a vasovagal syncopal event. The ethical aspects of this neurophysiology testing protocol were reviewed by the University of Minnesota Institutional Review Board (IRB), and formal IRB approval was deemed unnecessary for single-pulse assessment of healthy control participants not directly involved in a research study. Informed consent was obtained by the participant, including review of potential adverse events.

**Conclusion:**

Although rare and rarely reported, vasovagal syncopal events surrounding non-invasive brain stimulation do occur. Thorough pre-screening should incorporate assessment of history of syncope and a plan for risk mitigation if such an event should occur. A complete assessment of the impact of stimulation on the autonomic nervous system is unknown. As such studies expand into patients with myriad neurologic diagnoses, further studies on this effect, in both healthy control and patient populations, are warranted. Such knowledge could contribute to identification of the optimal study participant, and improvements in techniques of stimulation administration.

**Keywords:**

Non-invasive brain stimulation, Transcranial magnetic stimulation, Vasovagal syncope, Adverse events

## Background

Transcranial magnetic stimulation (TMS) involves the application of a transient magnetic field external to the skull, in single or repetitive fashion, to induce neuronal depolarization. Rarely, seizure activity has been reported in cases involving both single and repetitive-pulse TMS [1]. TMS-related syncopal events, or non-epileptiform loss of consciousness due to transient alteration of blood pressure and/or heart rate, have also been reported in rare instances [2, 3]. Vasovagal syncope (VVS), also referred to as reflex or neurally-mediated syncope, manifests as a transient loss of consciousness secondary a brief drop in systemic blood pressure and/or heart rate in response to emotional distress leading to global cerebral hypoperfusion [4]. We report a case of VVS associated with TMS in an otherwise healthy adult participant.

## Case presentation

A 47 year old, right-handed female was recruited as a healthy control participant to undergo TMS testing over the primary motor cortex. The participant reported medical history of hypothyroidism s/p partial thyroidectomy in 2007 with a daily 75 mcg tablet of oral levothyroxine as her only medication. No risk factors of prior closed head injury, loss of consciousness, history of seizure or febrile seizures, history of epilepsy or fainting were reported at the start of the TMS session. On the day of testing, the participant reported normal sleep, no changes in medication, no illicit drug use or usage of alcohol or caffeine.. The participant reported no symptoms at baseline on an IRB approved questionnaire.

### Transcranial magnetic stimulation

Baseline blood pressure and heart rate were 110/64 mmHg and 66 beats per minute, (bpm), respectively. She was positioned in 30° reclined seated position in a Brainsight TMS chair for TMS testing (Brainsight, Montreal, Canada). Surface Ag-AgCl bipolar recording electrodes were placed over the right hand first dorsal interosseous muscle after skin cleansing with an alcohol pad. A headband with stereotactic neuronavigation tracking software was applied to forehead with no discomfort reported by the participant.

Single-pulses of TMS were delivered using a Magstim Bistim^2^ and 200^2^ set with a 70 mm, figure-eight coil (Magstim Company Ltd., Dyfed, UK). The location of the left cortical motor threshold was determined by placing the coil 45° from perpendicular over the posterior frontal head region at the primary motor cortex hotspot for hand representation. Criteria for localizing the motor hotspot was determined through obtaining a motor evoked potential of at least 50 uV in amplitude on 3/5 trials using a Motion-Lab electromyography system (Y03-2, Motion Lab Systems, Inc., Baton Rouge, LA, USA) with custom made LabView software (v2012, National Instruments, Austin, TX, USA). The resting motor threshold was determined to be at 50 % machine maximum stimulus intensity. The participant reported no discomfort during resting motor threshold testing. The testing proceeded to obtain 1 mV motor evoked potentials with TMS over the left motor hotspot starting with stimulus intensity 120 % of the resting motor threshold (60 % machine maximum). Stimulus intensity was then increased to 65, 67 and 70 % of machine maximum with stimulus intervals of ≥20 s.

### Syncopal event

Approximately thirty minutes into the testing session and upon the 128^th^ pulse on the left hemisphere, the participant reported feeling “queasy”. Testing was immediately stopped and the participant was offered a glass of water. The participant then reported feeling “dizzy” and the water was removed from her hand. Two minutes after the last magnetic stimulation, the participant appeared to have fainted. The TMS operator noted that the participant’s face was pale for 10 s prior to loss of consciousness. The participant turned her face to the left with her eyes remaining closed. Her left arm assumed a flexed posture with what appeared to be myoclonic type jerking for 2–3 s in duration. Jerking activity was also noted in the right arm but not to the same intensity. No motor movements were noted involving the lower extremities. All electrodes were detached, and when the jerking movements of the upper extremities ceased, the participant was reclined to the full horizontal position. After cessation of the jerking movements, the participant’s eyes remained closed and responded “yes” to the TMS operator question on whether or not she could hear the operator. In the reclined position, and approximately nine minutes after loss of consciousness, her blood pressure and pulse were recorded as 110/61 mmHg and 61 bpm respectively. The participant reported that when the symptoms started, she felt that she was “not seeing clearly” and “started to get warm”. She did not have any urinary or fecal incontinence, nor post-event confusion or trauma. The participant felt she could ambulate and the session was ended.

### Post-event assessment

The participant was then escorted to an urgent care clinic, where a mental status, general and neurologic examination were performed by an internal medicine physician. Orthostatic vital sign testing was normal with supine and immediate standing blood pressure and pulse of 108/74 mmHg, 78 bpm and 123/86 mmHg, 95 bpm, respectively. Cardiovascular and neurologic examinations were noted to be normal. The participant reported normal intake of food and water on this day, and the evaluating physician did not conclude it necessary to perform a blood glucose test. The participant noted upon specific questioning of “have you personally ever fainted?” instead of “do you have a history of fainting?” to have had a single syncopal episode, approximately 10 years ago, while donating blood. The participant stated that her interpretation of our original questioning “do you have a history of fainting?” pertained to a family history, not personal, history of fainting events. The urgent care physician determined the diagnosis as VVS based on past history of syncope, absence of fecal or urinary continence, full recall of the event without confusion, and stability of vital signs on orthostatic assessment. Based on the diagnosis of VVS, the physician determined that serum chemistries, electroencephalography and brain imaging were not indicated. The electrophysiology laboratory and urgent care notes were further reviewed by the study neurologist who concurred with the diagnosis of VVS. At one, four and twelve-week phone follow-up with the participant, no further symptoms were reported.

## Conclusions

VVS may occur rarely in association with TMS, but appears to be a benign symptom without delayed morbidity. Myoclonic muscle jerking has been reported in up to 27 % of subjects with observed VVS, and it is important not to confuse this motor phenomenon with seizure activity, as VVS is felt to be a more benign clinical entity generally requiring no further laboratory investigation [5, 6]. VVS events occur more often in younger individuals and women, and attacks are commonly triggered by anxiety, noxious stimuli, prolonged standing and increased thoracic pressure [6]. Interestingly, our participant reported no anxiety or discomfort with the TMS procedure prior to loss of consciousness. Therefore, it is unclear as to whether this episode of VVS was secondary to an emotional response or direct effect of TMS on autonomic nervous system function. We recommend that participants undergoing TMS should be screened thoroughly for any history of syncopal events prior to undergoing the procedure. Additionally, creating screening questionnaires which are readily understood by the population studied can elucidate and detail a more complete medical history. Such information will allow consideration of any/all medical diagnoses and medications (i.e.-thyroid hormones and centrally-acting medications) which may influence cortical excitability and the potential for syncopal or seizure activity [7, 8]. Real-time integration of TMS with electroencephalography (EEG) is also recommended for surveillance of underlying ictal brain activity during TMS administration [1]. Further studies of the effects of TMS on autonomic nervous system function are also warranted to investigate the impact of non-invasive brain stimulation interventions in populations with and without specific history of autonomic nervous system dysfunction.

## Consent

Written informed consent was obtained from the participant for publication of this Case report and any accompanying images. A copy of the written consent is available for review by the Editor of this journal.

## Abbreviations

TMS: Transcranial magnetic stimulation
VVS: Vasovagal syncope
